# Rotating-pulling-poking manipulation effectively alleviates pain symptoms of lateral ankle sprain: an animal experimental study

**DOI:** 10.3389/fpain.2025.1532867

**Published:** 2025-06-23

**Authors:** Haibao Wen, Jinghua Gao, Fudong Shi, Jin Li, Minrui Fu, Minshan Feng, Luguang Li, Chunyu Gao, Jianguo Li

**Affiliations:** ^1^ Wangjing Hospital, China Academy of Chinese Medical Sciences, Beijing, China; ^2^ Beijing Key Laboratory of Mathematical Wisdom and Traditional Chinese Medicine for the Prevention and Treatment of Bone and Joint Degenerative Diseases, Beijing, China; ^3^ Foshan Hospital of Traditional Chinese Medicine, Foshan, Guangdong, China; ^4^ The Eighth Clinical Medical College of Guangzhou University of Chinese Medicine, Foshan, China

**Keywords:** rotating-pulling-poking manipulation, ankle lateral sprain, analgesic mechanisms, biological effects, animal experimental

## Abstract

This study aimed to explore the analgesic mechanism of the Rotating - Pulling - Poking Manipulation in treating acute lateral ankle sprain (ALAS). Thirty rats were randomly divided into 5 groups in the first experiment to determine the dose - effect relationship by detecting ankle pain thresholds at different time points. The results showed that the 5-min manipulation group had the best analgesic effect, with the bipedal weight - bearing difference decreasing over time and pain relief time shortened. In the second experiment, 30 rats were divided into 3 groups. After manipulation, samples from PAG and RVM were tested. The results indicated that compared with the model group, the 5-min manipulation group had increased MOR expression in PAG and 5 - HT concentration in cerebrospinal fluid, decreased expressions of BDNF, p - TrkB/TrkB in PAG and NR2A in RVM, and reduced contents of IL - 6, IL - 1β, TNF - α in ankle soft tissues. In conclusion, the Rotating - Pulling - Poking Manipulation can relieve pain by exciting the descending inhibitory system mediated by opioid receptors in the brain center, weakening the descending facilitation system mediated by the BDNF/TrkB/NR2A signaling pathway, and reducing the inflammatory response.

## 1 Introduction

Acute lateral ankle sprain (ALAS)-induced pain falls under the category of inflammatory pain (1). After ALAS occurs, the lateral ankle tissues are subjected to tension (2), resulting in damage to structures such as ligaments, muscles, and blood vessels (3). This triggers alterations in the local biochemical and metabolic environment, including sterile inflammatory exudation, release of inflammatory factors, increased vascular permeability, and oxidative stress (4). These changes activate nociceptors at the injury site, opening associated ion channels and generating action potentials (5). These action potentials are transmitted from the periphery to the spinal cord via A-delta or C nerve fibers in the dorsal root ganglion (DRG) and further processed in the spinal dorsal horn (Rexed laminae) and ascending pathways to the somatosensory cortex, resulting in the perception, intensity, and localization of pain (6). This constitutes the ascending transmission pathway of acute pain.

Pain processing is also subject to descending modulation from higher spinal and supraspinal centers, primarily involving descending inhibitory and facilitation systems. Activation of the descending inhibitory system reduces pain sensitivity, playing a role in endogenous analgesia by limiting peripheral nociceptive input. Conversely, activation of the descending facilitation system enhances sensitivity to peripheral nociceptive stimuli, leading to hyperalgesia (7). Dysregulation of these systems may result in abnormal pain perception (8). The descending pain modulation system operates through the periaqueductal gray (PAG), rostral ventromedial medulla (RVM), and spinal dorsal horn (SDH), forming the PAG-RVM-SDH pain modulation pathway.

Research has demonstrated that the endogenous opioid peptide system plays a key role in the descending inhibitory system (9). Opioid-peptidergic neurons release opioid peptides that bind to opioid receptors, inhibiting GABAergic and glutamatergic neurons in the PAG. This activation directly stimulates serotonergic neurons in the SDH, releasing the neurotransmitter serotonin (5-HT), thereby exerting endogenous analgesic effects (10, 11). The opioid receptors involved include μ-opioid receptor (MOR), δ-opioid receptor (DOR), and κ-opioid receptor (KOR).The activation of the descending facilitation system is associated with the brain-derived neurotrophic factor (BDNF)/tropomyosin receptor kinase B (TrkB)/N-methyl-D-aspartate receptor 2A (NR2A) signaling pathway, which plays a role in pain facilitation. During peripheral inflammatory pain, the expression of BDNF in the PAG is significantly increased, binding to TrkB receptors on neuronal membranes to form ligand-receptor complexes. This initiates TrkB phosphorylation and enhances NR2A receptor activity and expression through downstream intracellular signaling cascades, providing evidence of increased activation of facilitation-related receptors (12–14).

Evidence-based clinical trials have shown that rotating-pulling-poking manipulation effectively alleviates pain in ALAS, demonstrating favorable clinical outcomes (15, 16). However, the underlying mechanisms of this intervention remain incomplete. This study aims to confirm that the rotating-pulling-poking manipulation can modulate the descending inhibitory system mediated by central opioid receptors and attenuate the descending facilitation system mediated by the BDNF/TrkB/NR2A signaling pathway through mechanical stimulation. By doing so, it effectively alleviates pain symptoms in ALAS rats, providing experimental and theoretical evidence for the analgesic mechanisms of rotating-pulling-poking manipulation in ALAS treatment.

## 2 Methods

### 2.1 Experimental animals

A total of 60 healthy adult male Sprague Dawley (SD) rats of SPF grade (Female rats were excluded due to their 4–5-day estrous cycle, during which estrogen levels fluctuate cyclically, potentially confounding neurobehavioral assessments), aged 9 weeks, and weighing approximately 230–250 g were selected. The animals were procured from SPF Biotechnology Co., Ltd., Beijing [animal production license: SCXK (Beijing) 2022-0002; animal quality certificate: NO. 111251220100136723]. They were housed in the animal facility of the Institute of Basic Theory, China Academy of Chinese Medical Sciences, under controlled conditions (temperature 20°C–25°C, humidity 50%–65%, and a 12-hour light-dark cycle) with free access to food and water.

### 2.2 Experimental instruments and equipment

(1) Flexible thin-film pressure sensor: Model DF-940, Suzhou Nengsida Electronic Technology Co., Ltd.; (2) Thin-film pressure sensor detection module: Model MY2801, Suzhou Nengsida Electronic Technology Co., Ltd.; (3) Plantar Test Apparatus: Model IITC600, Life Science Inc., USA; (4) Pathological microtome: Model RM2016, Leica Microsystems Shanghai Ltd.; (5) Embedding machine: Model JB-L5, Wuhan Junjie Electronics Co., Ltd.; (6) Tissue slide dryer: Model KD-P, Kedi Instruments Co., Ltd., Zhejiang.

### 2.3 Reagents

DOR antibody, MOR antibody, 5-HT ELISA kit, IL-6 antibody, IL-1β antibody, TNF-α antibody, BDNF rabbit polyclonal antibody, TrkB rabbit polyclonal antibody, p-TrkB rabbit polyclonal antibody, NR2A rabbit monoclonal antibody, beta-Actin monoclonal antibody (Servicebio, Models BS-3624R, BS-1094R, BS-3623R, CEA808GE, GB11117, GB11113, GB11188, GB11559, GB11295-1, ab197072, ab124913, GB15001).

### 2.4 Model establishment procedures

Sodium pentobarbital solution (0.3%) was intraperitoneally injected at a dose of 10 ml/kg. Anesthesia was confirmed when the rats showed no pain reflex to limb pinching and no corneal reflex. Modeling method (17): after anesthesia, rats were placed in a left lateral position. The operator used the left hand to secure the distal right lower limb and the right hand to hold the ankle joint using the thumb, index, middle, and ring fingers, with the thumb placed on the lateral margin of the ankle. The procedure was as follows: (1) For 1 min, the right hind limb's ankle joint was bent repeatedly in a plantarflexion-inversion direction at a rate of approximately 1 motion per second until the joint could flex from a resting position to a 90° inversion; (2) Subsequently, the inversion was repeated for 1 min until the joint could flex to a 180° inversion from a resting position; (3) Steps 1 and 2 were repeated once, with the total modeling time being approximately 4 min.

## 3 Experiment one

### 3.1 Experimental grouping

Thirty rats were randomly divided into 5 groups, with 6 rats per group, and subjected to different interventions:

(1) Blank control group: no intervention or treatment;
(2) Model control group: ALAS model established with no intervention;
(3) 1-min Rotating-Pulling-Poking Manipulation Group in ALAS Model Rats;
(4) 5-min Rotating-Pulling-Poking Manipulation Group in ALAS Model Rats;
(5) 10-min Rotating-Pulling-Poking Manipulation Group in ALAS Model Rats.

### 3.2 Rotating-pulling-poking manipulation intervention

#### 3.2.1 Pre-intervention preparation

To ensure consistency in force and frequency during the rotating-pulling-poking manipulation, a flexible thin-film pressure sensor (5 kg range) was used to monitor the force and frequency exerted by the operator's thumb in real-time. The sensor displayed the applied force and generated a force-time curve, with parameter data simultaneously exported to a computer for storage. Before manipulation, the pressure sensor was secured to the operator's thumbpad using tape. The intervention followed principles from previous studies on animal manual manipulation (18), where a dedicated operator performed the procedure at a fixed time (9:00 AM–12:00 PM).

#### 3.2.2 Manipulation procedure

**Figure 1:**
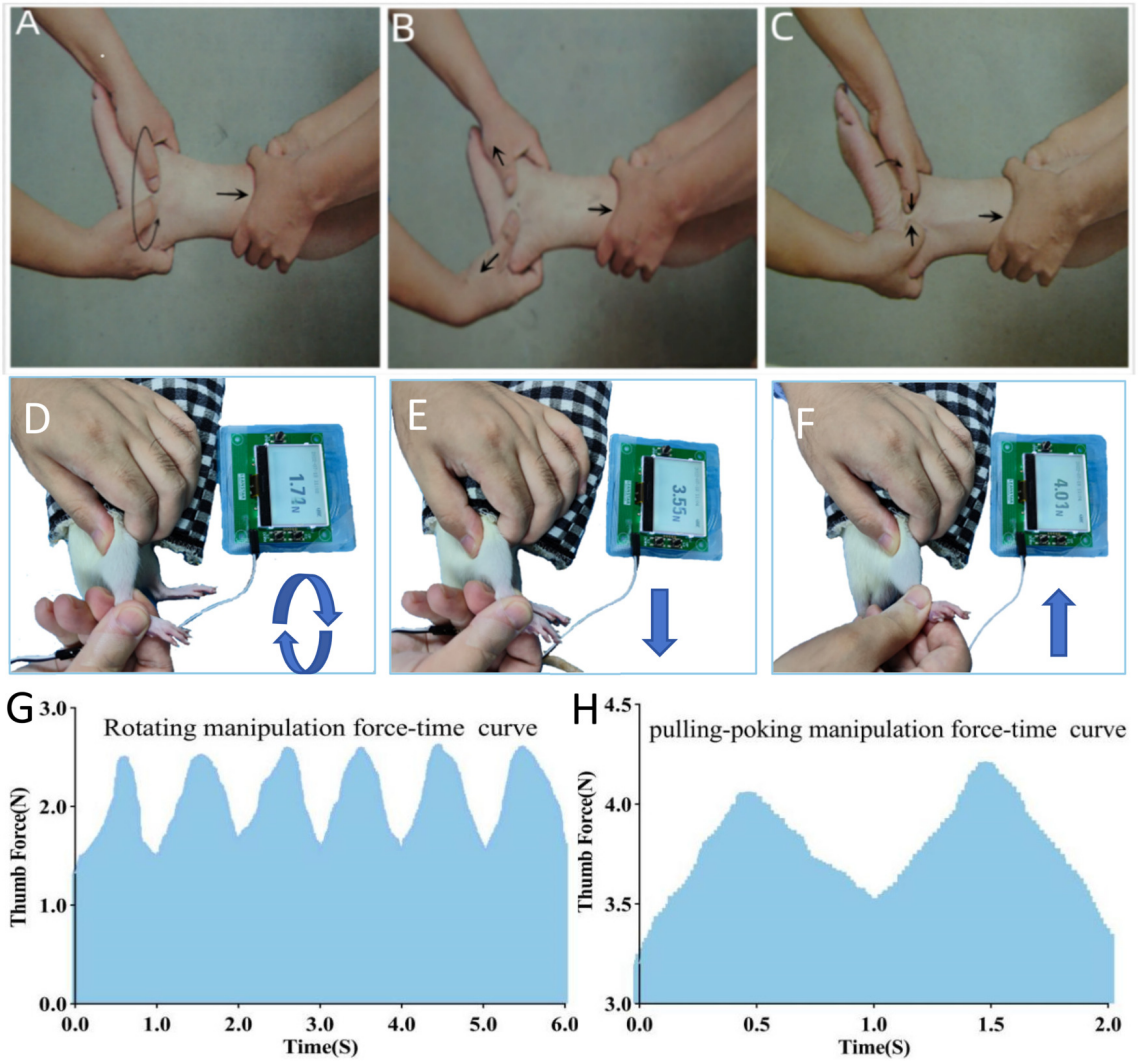
Illustrates the procedure: **(A,D)** rotation manipulation; **(B,E)** pulling manipulation; **(C,F)** poking manipulation; **(G,H)** force-time curve during manipulation.

The magnitude of force during manipulation depended on the rats’ tolerance. Rats were considered to have good tolerance if there were no evident limb retraction, escape behaviors, or vocalization during the procedure (19). Based on this principle, optimal force-time curves and parameter values for the rotating-pulling-poking manipulation in ALAS rats were determined (Figures 1G,H, Table 1). These values were used as the reference standard for the formal experiment.

**Table 1:** Thumb force (*N*) for rotating-pulling-poking manipulation on rats.

Manipulation	Mean ($\bar{X}$)	Standard deviation (SD)	Maximum (max)	Minimum (min)
Rotating	2.06	0.35	2.63	1.32
Pulling	3.72	0.21	4.06	3.20
Poking	3.81	0.23	4.21	3.35

The procedure for rotating-pulling-poking manipulation consisted of three steps: (1) The operator placed the thumbpad of the right hand, equipped with a fixed pressure sensor, on the swollen area of the lateral ankle while supporting the medial ankle with the index and middle fingers. The ankle was rotated at approximately 1 cycle per second, completing six rotations (three clockwise and three counterclockwise) (Figures 1A,D). (2) The ankle joint was then gently pulled and extended while dorsiflexing and inverting it until mild resistance was felt from the soft tissue, pausing for about 1 s (Figures 1B,E). (3) The ankle joint was subsequently plantarflexed and everted until mild resistance was felt, during which the operator's thumb applied a downward poking motion on the lateral ankle's affected area (Figures 1C,F). These steps were repeated, ensuring the manipulation was smooth, orderly, gentle, and precise.

#### 3.2.3 Frequency of manipulation intervention

**Figure 2:**
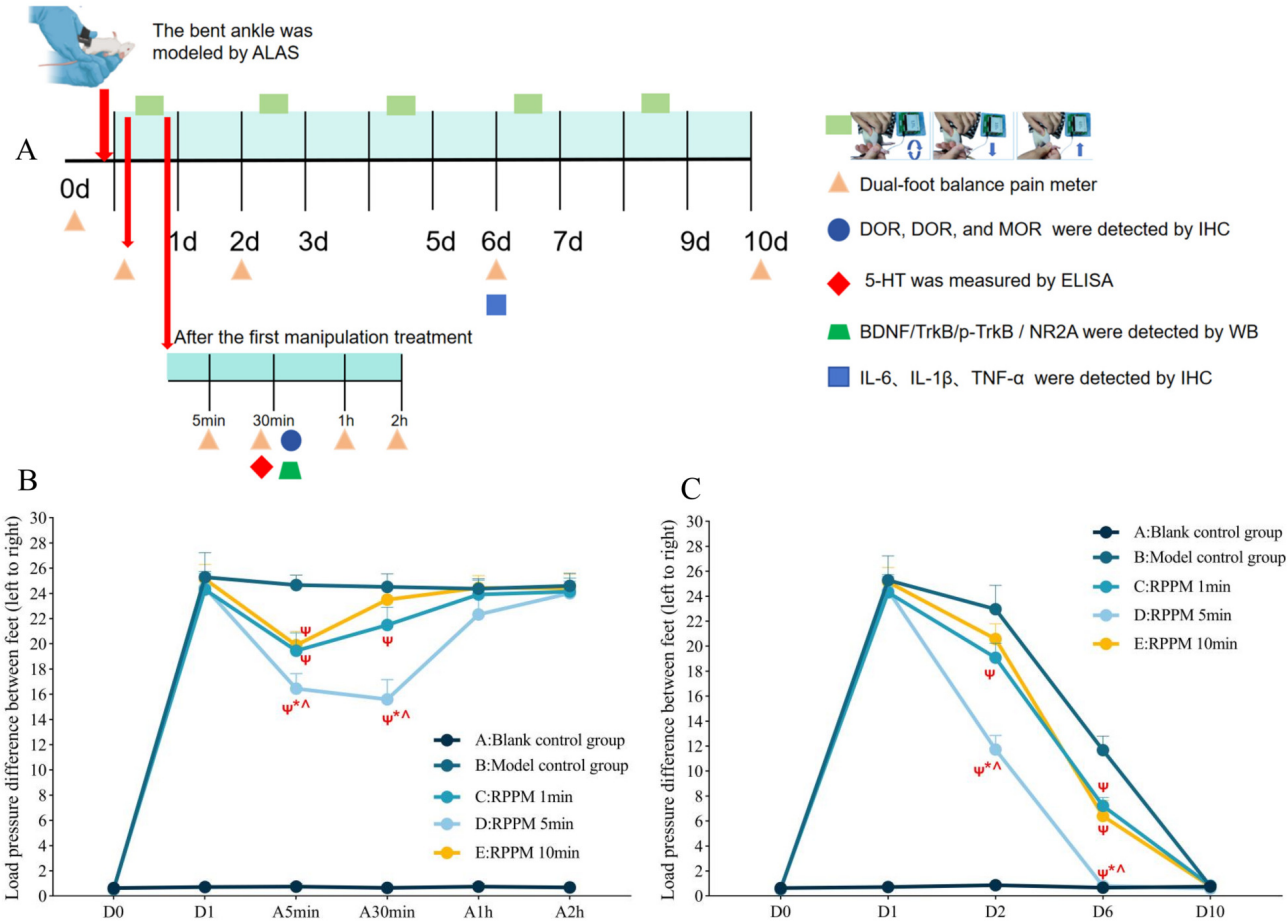
(Experiment One): Time points for bilateral foot balance measurement and analgesic mechanism-related indicator testing in ALAS rats treated with the rotating-pulling-poking manipulation. **(A)** Flowchart of detection time points; **(B,C)** Measurement results of bilateral foot loading pressure differences in rats. Ψ*P* < 0.05 vs. **B**; *P < 0.05 vs. **C**; #*P* < 0.05 vs. **D**; ^*P* < 0.05 vs. **E**; A5min (5 min after manipulation therapy), A30min (30 min after manipulation therapy), A1h (1 h after manipulation therapy), and A2h (2 h after manipulation therapy).

Rotating-pulling-poking manipulation therapy was initiated on the first day post-modeling and administered every other day for a total of 5 sessions. Treatments were conducted on days 1, 3, 5, 7, and 9 post-modeling, with durations of 1, 5, or 10 min depending on the group assignment (Figure 2A).

### 3.3 Measurement indicators

#### 3.3.1 Ankle pain assessment

Ankle pain assessment in rats was conducted at two stages: first, measurements were taken before modeling (D0), on the first day post-modeling (before manipulation), and 5 min, 30 min, 1 h, and 2 h after the first manipulation to determine whether the rotating-pulling-poking manipulation had an immediate analgesic effect. Pain threshold measurements were also taken on days 1, 2, 6, and 10 post-modeling to evaluate whether the manipulation shortened the pain duration in ALAS rats (Figure 2A).

Prior to modeling, a 3-day acclimatization training was conducted to improve behavioral testing compliance. Pain assessments were carried out by two blinded experimenters. The ankle pain level was measured using a bilateral balance pain tester, which indirectly reflects pain severity based on the rats’ foot loading during standing. Heavier loading indicates less pain, while lighter loading indicates more severe pain (20). Procedure: Rats were placed in a test chamber, with forelimbs resting on an inclined panel and hindlimbs standing, ensuring each foot was on a separate pressure sensor plate. The tail was kept outside the chamber to avoid weight measurement errors. During testing, pressure values of the left and right feet were recorded continuously for 10 s. Valid data for each rat were collected three times, and the difference in foot loading between the affected and unaffected sides was calculated (21). The average of the three measurements was used as the quantified pain result. The parameters for bilateral hindlimb balance algometry were set as follows: single measurement duration 60s, sensor resolution 0.01 g, pedal angle 75°, and weight-bearing difference threshold 20%.

## 4 Experiment two

### 4.1 Animal grouping

Based on the results of Experiment One, the 5-min intervention group showed superior efficacy in alleviating ankle pain in ALAS rats compared to the 1-min and 10-min groups. Thus, 5 min was determined to be the optimal intervention duration for the rotating-pulling-poking manipulation in ALAS rats. A 5-min intervention significantly alleviated pain symptoms by day 6 post-modeling; therefore, interventions in this experiment were conducted on days 1, 3, and 5 post-modeling. Thirty rats were sequentially numbered from 01 to 30 and randomly divided into three groups (10 rats per group) using a random number table generated in Excel: (1) Blank Control Group: No interventions performed. (2) Model Control Group: ALAS modeling was successfully established, but no interventions were performed. (3) 5-min Rotating-Pulling-Poking Manipulation Group in ALAS Model Rats: Interventions were performed between 9:00 a.m. and 12:00 p.m. on days 1, 3, and 5 post-modeling, with each session lasting 5 min.

### 4.2 Measurement indicators

#### 4.2.1 Immunohistochemical detection of DOR, DOR, and MOR in PGA sections, and Il-6, Il-1β, and TNF-α in ankle soft tissue

Thirty minutes after the manipulation intervention, three rats were randomly selected from each group. These rats were intraperitoneally anesthetized with 0.3% sodium pentobarbital, after which their thoracic cavity and heart were exposed. Cardiac perfusion was performed with 0.9% saline and 4% paraformaldehyde until the rats became rigid. Tissue samples were collected from the periaqueductal gray area (PGA), dehydrated, and embedded in paraffin. On day 6 post-modeling, three rats from each group were randomly selected, deeply anesthetized, and prepared for surgical removal of the right ankle joint. The joint was carefully excised 0.5–1 cm around the ankle to obtain intact ankle joint soft tissue for analysis.

Processing Steps: Tissue sections were deparaffinized and rehydrated in water. Primary antibody (1:1,000) was applied, and the samples were incubated overnight at 4°C. Secondary antibody (1:200) was added and incubated at room temperature for 1 h. DAB chromogenic reaction was performed by mixing 50 µl each of reagents A, B, and C from the DAB staining kit with 0.85 ml distilled water. A 100 µl solution was applied to the sections for 3 min. Hematoxylin staining was conducted for 45 min, followed by dehydration and mounting with neutral resin. Protein expression was evaluated under a bright-field microscope, and the average optical density under 200× magnification was quantified using Image J 1.50.

#### 4.2.2 Western blot detection of BDNF, TrkB, p-TrkB, and Nr2a protein expression in PAG and RVM

Thirty minutes after the manipulation intervention, four rats from each group were randomly selected. Protein expression of BDNF, TrkB, p-TrkB in the PAG and NR2A in the RVM was detected using Western blot. This analysis was performed in the normal, model, and manipulation groups to identify differences in protein expression among the groups, following the instructions of the Western blot kit.

#### 4.2.3 ELISA for determination of 5-HT levels in cerebrospinal fluid

Thirty minutes after the manipulation intervention, four rats from each group were randomly selected and intraperitoneally anesthetized with 0.3% sodium pentobarbital. After preparing the occipital region, rats were placed in a prone position and fixed on a rat board. A longitudinal incision was made along the midline of the posterior occipital region to expose the atlanto-occipital membrane. The incision was irrigated with 0.9% saline to remove clots and dried with clean gauze, ensuring no active bleeding and a clear surgical field. A micropipette was prepared to puncture the atlanto-occipital membrane gently, inserting approximately 1–2 mm into the subdural space. Cerebrospinal fluid was collected in small increments into centrifuge tubes, and 5-HT levels were measured following the instructions in the ELISA kit.

### 4.3 Statistical analysis

Statistical analyses and graph plotting were performed using SPSS 25.0 and GraphPad Prism 8.0. All quantitative data were first subjected to the Shapiro–Wilk test for normality. Normally distributed data were described using mean ± standard deviation (SD) for central tendency and dispersion, while non-normally distributed data were expressed as median (first quartile, third quartile). For multi-group comparisons of the same variable, either one-way ANOVA (for parametric data) or the Kruskal–Wallis *H*-test (for non-parametric data) was applied. Repeated measures ANOVA was utilized for within-group comparisons across time points (e.g., behavioral assessments at baseline and post-intervention). Pairwise comparisons between groups at specific time points were conducted using Tukey's *post hoc* test under the one-way ANOVA framework. The significance level was set at *α* = 0.05, and a two-tailed *P* < 0.05 was considered statistically significant.

## 5 Results

### 5.1 Measurement of ankle pain behavior in rats

As shown in Figure 2B, 5 min after the manipulation treatment, the difference in bilateral foot loading in groups C, D, and E was significantly lower than that in the model group (*P* < 0.001). Group D had a smaller bilateral foot loading difference compared to groups C and E (*P* < 0.05). Thirty minutes after treatment, groups C and D still showed significantly lower bilateral foot loading differences than the model group (*P* < 0.001), while group E showed no statistically significant difference from the model group (*P* > 0.05). Group D had a smaller bilateral foot loading difference compared to groups C and E (*P* < 0.001). At 1 and 2 h post-treatment, no statistically significant differences were observed among the groups (*P* > 0.05).

As shown in Figure 2C, on day 2 post-modeling, the bilateral foot loading difference in groups C and D was significantly lower than that in the model group (*P* < 0.05), while no statistically significant difference was observed between group E and the model group (*P* > 0.05). Group D exhibited a smaller bilateral foot loading difference compared to groups C and E (*P* < 0.001). On day 6 post-modeling, groups C, D, and E showed significantly lower bilateral foot loading differences compared to the model group (*P* < 0.001). Group D again showed a smaller bilateral foot loading difference than groups C and E (*P* < 0.001). On day 10 post-modeling, no statistically significant differences were found among the groups (*P* > 0.05).

### 5.2 Expression of MOR, KOR, and DOR in the PAG of rat brains

**Figure 3:**
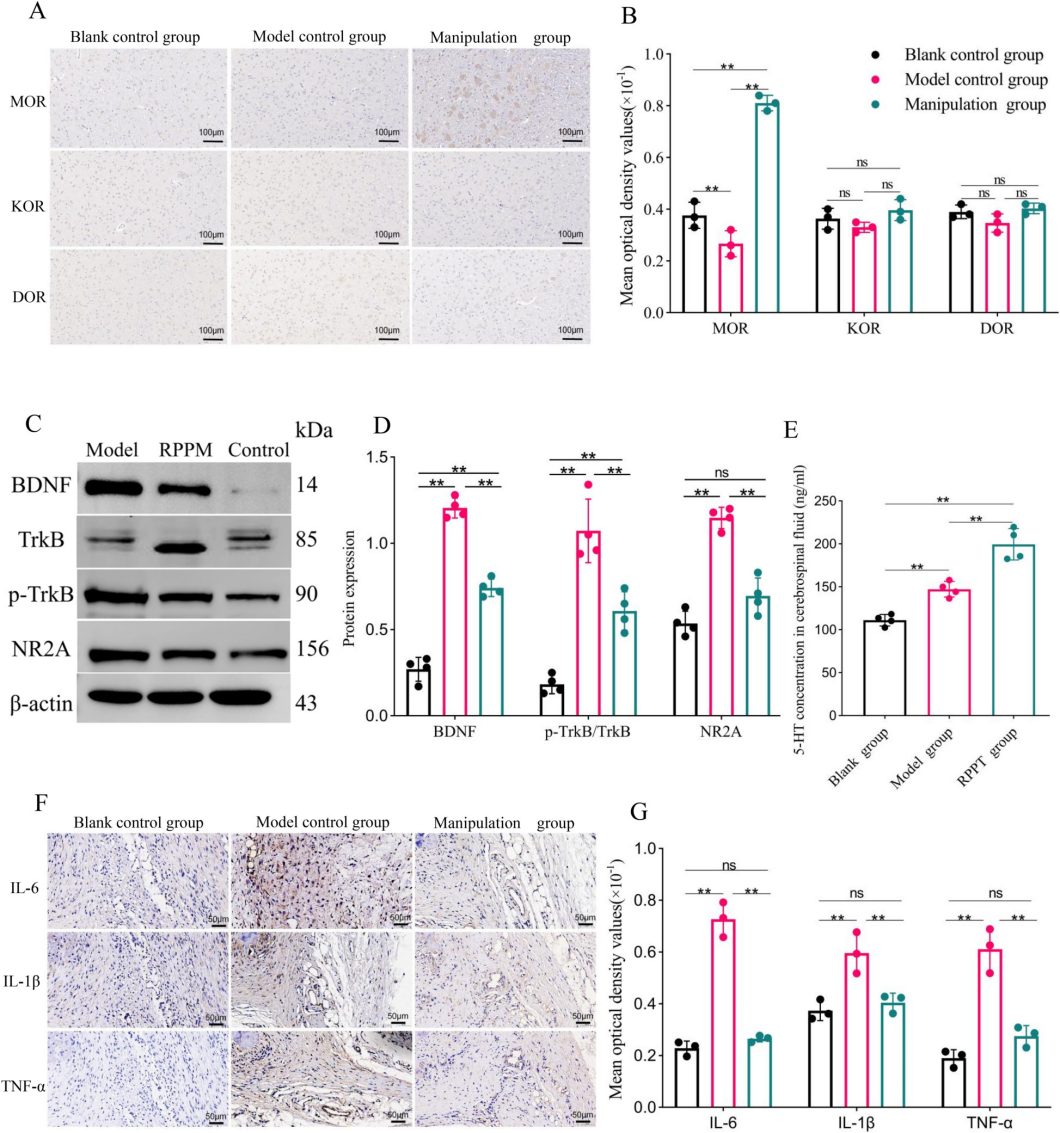
(Experiment Two): **(A,B)** results and comparisons of MOR, KOR, and DOR expression in the PAG of rats (X ± S, *n* = 3); **(C,D)** results and comparisons of BDNF, trkB, p-trkB expression in the PAG and NR2A expression in the RVM of rats (X ± S, *n* = 4); **(E)** comparison of 5-HT concentration in rat cerebrospinal fluid (X ± S, *n* = 4, ng/ml); **(F,G)** results and comparisons of IL-6, IL-1β, and TNF-α expression in rat ankle joint soft tissue (X ± S, *n* = 3). ns: *P* > 0.05; *: *P* < 0.05; **: *P* < 0.001.

As shown in Figures 3A,B, the molecular expression of MOR in the model group was significantly lower than in the blank control group (*P* < 0.01). After treatment with the rotating-pulling-poking manipulation, the expression of MOR in the manipulation group was significantly higher than in the blank control group (*P* < 0.01). No statistically significant differences in the expression of KOR and DOR were observed among the groups (*P* > 0.05).

### 5.3 Expression of BDNF, TrkB, p-TrkB, and Nr2a proteins in the PAG and RVM of rats

As shown in Figures 3C,D, the expression levels of BDNF and p-TrkB/TrkB proteins in the PAG of the model control group were higher than those in the blank control group (*P* < 0.01). Similarly, the expression level of NR2A protein in the RVM was also higher in the model control group than in the blank control group (*P* < 0.01). After treatment with the rotating-pulling-poking manipulation, the expression levels of BDNF and p-TrkB/TrkB proteins in the PAG, as well as NR2A protein in the RVM, were significantly lower in the manipulation group compared to the model control group (*P* < 0.01). Although BDNF levels and the p-TrkB/TrkB ratio in the manual therapy group were reduced to levels significantly lower than those in the model control group (*P* < 0.05), they remained higher than those in the blank control group (*P* < 0.05).

### 5.4 5-HT concentration in rat cerebrospinal fluid

As shown in Figure 3E, the concentration of 5-HT in the cerebrospinal fluid of the modeled rats was significantly higher than that of the blank control group (*P* < 0.01). After treatment with the rotating-pulling-poking manipulation, the concentration of 5-HT in the cerebrospinal fluid of the manipulation group was further increased, showing a statistically significant difference compared to the model control group (*P* < 0.001).

### 5.5 Protein expression of Il-6, Il-1β, and TNF-α in ankle joint soft tissue

As shown in Figures 3F,G, on day 6 post-modeling, the expression levels of IL-6, TNF-α, and IL-1β in the model control group were significantly higher than those in the blank control group (*P* < 0.001). After treatment with the rotating-pulling-poking manipulation, the expression levels of IL-6, TNF-α, and IL-1β in the manipulation group were significantly lower than those in the model control group (*P* < 0.001).

## 6 Discussion

The therapeutic effects of manipulation may be mediated by the descending modulation pathways, which constitute a primary mechanism of the analgesic effects of manual therapy (22). Activation of the descending inhibitory system is a common focus in studies of the immediate analgesic mechanisms of manipulation (23, 24). Therefore, this study preliminarily explored whether the rotating-pulling-poking manipulation exerts immediate analgesic effects by modulating the dynamic balance of the descending modulation system from the perspectives of descending inhibition and facilitation.

In Experiment 1, The rotating-pulling-poking manipulation demonstrated immediate analgesic effects on ankle pain behaviors in ALAS rats, with the effect being most pronounced 30 min after the intervention. In Experiment 2, 30 min after the manipulation, MOR expression in the vlPAG region of the brain was significantly increased in the treatment group, while it was reduced in the model control group. Additionally, the concentration of 5-HT in the cerebrospinal fluid of the manipulation group was markedly elevated. Studies have shown that the vlPAG and MOR are the most prominent regions and receptors of the endogenous opioid system involved in pain regulation, and this descending regulatory pathway is closely associated with 5-HT (25, 26). Skyba DA et al. also found that joint manipulation activated the descending inhibitory pathway and generated neurotransmitter 5-HT, which acted on 5-HT1A receptors in the spinal cord to inhibit the transmission of nociceptive signals (26). Santos FM et al. investigated the analgesic effects of manipulation from the perspective of descending pain inhibition. They reported a 17% increase in KOR expression in the PAG of rats with chronic nerve compression injury following knee joint manipulation, while MOR expression showed no significant change. Santos FM suggested that different animal pain models may influence the expression of various opioid receptors, which could explain the discrepancies between their results and those of this study (27). This study found that the expression of BDNF, p-TrkB/TrkB in the PAG and NR2A in the RVM was lower in the manipulation group compared to the model control group. The rotating-pulling-poking manipulation suppressed the BDNF/TrkB/NR2A signaling pathway, which is associated with the facilitative effects of the descending pain modulation system (13).

Additionally, in Experiment 2, we found that the rotating-pulling-poking manipulation not only exhibited immediate analgesic effects but also effectively shortened the duration of ankle pain in ALAS rats, suggesting that alternate-day interventions with this technique could alleviate pain in ALAS rats. In this study, we used immunohistochemistry (IHC) to assess the levels of inflammatory cytokines IL-6, IL-1β, and TNF-α in the lateral ankle soft tissue of rats. On day 6 after modeling (following three manipulation interventions), the levels of these inflammatory cytokines were found to be lower in the manipulation group compared to the model group. Studies have indicated that IL-6, IL-1β, and TNF-α are pro-inflammatory cytokines most closely associated with the exacerbation of pain (28). Acute peripheral inflammatory pain is linked to the infiltration of immune cells at the injury site. After tissue damage, the release of inflammatory mediators and the acidic chemical environment of the tissue activate primary sensory neurons, lowering their threshold and inducing hyperalgesia (29). Relevant scholars suggest that manual therapy, as a mechanical intervention, has immunomodulatory effects (30). Altering the expression levels of local inflammatory mediators is considered a primary mechanism of the peripheral analgesic effects of orthopedic manipulations (31, 32), which aligns with the findings of this study.

Following lateral ankle injury, locally released pro-inflammatory cytokines such as IL-6, IL-1β, and TNF-α directly activate peripheral nociceptors (e.g., TRPV1 and P2X3 receptors) (4). The resultant nociceptive signals are transmitted via Aδ/C fibers through the spinal dorsal horn to supraspinal regions including the periaqueductal gray (PAG) and rostral ventromedial medulla (RVM), triggering central sensitization (manifested as BDNF/TrkB/NR2A pathway activation in the PAG) (1). This mechanism aligns with the ascending nociceptive pathways underlying persistent pain in clinical ALAS patients.

The mechanical stimulation induced by the Rotating-Pulling-Poking (RPP) manipulative technique activates μ-opioid receptors (MOR) in the PAG through somatosensory afferent nerves, thereby disinhibiting GABAergic suppression of 5-hydroxytryptamine (5-HT) neurons in the RVM (10). This leads to a significant elevation in cerebrospinal fluid 5-HT concentrations. Previous studies demonstrate that 5-HT suppresses glutamatergic synaptic transmission via spinal 5-HT1A receptors (33), while concurrently activating the spinal-sympathetic nerve pathway to inhibit pro-inflammatory mediator release (e.g., IL-6) from peripheral macrophages via β2-adrenergic receptors. These findings are consistent with the observed reduction in IL-6 and TNF-α levels in the ankle tissues of the RPP intervention group. Notably, central 5-HT system activation not only suppresses nociceptive signaling (descending inhibition) but also attenuates peripheral neurogenic inflammation through neuro-immune crosstalk. This bidirectional regulatory mechanism forms a “peripheral inflammation→central sensitization→descending inhibition→peripheral anti-inflammatory” feedback loop (34), which may underlie the RPP technique's capacity to shorten the pain duration in ALAS from approximately 10 days to 6 days.

## 7 Conclusion

Therefore, we propose that the mechanism of the immediate analgesic effect of the rotating-pulling-poking manipulation is related to the regulation of the dynamic balance of the central descending modulation system. This manipulation can stimulate the descending inhibitory system mediated by central opioid receptors in the brain of ALAS rats while attenuating the descending facilitatory system mediated by the BDNF/TrkB/NR2A signaling pathway. This dual modulation alleviates the imbalance between descending inhibition and facilitation in the early stages of ALAS-induced pain, thereby reducing pain. Moreover, the rotating-pulling-poking manipulation can reduce the local expression levels of inflammatory mediators in the lateral ankle soft tissue of ALAS rats, which may be one of the mechanisms through which it effectively shortens the duration of ankle pain in ALAS rats.

## Data Availability

The original contributions presented in the study are included in the article/Supplementary Material, further inquiries can be directed to the corresponding authors.
